# Acceptance and Use of Home-Based Electronic Symptom Self-Reporting Systems in Patients With Cancer: Systematic Review

**DOI:** 10.2196/24638

**Published:** 2021-03-12

**Authors:** Youmin Cho, Huiting Zhang, Marcelline Ruth Harris, Yang Gong, Ellen Lavoie Smith, Yun Jiang

**Affiliations:** 1 University of Michigan School of Nursing Ann Arbor, MI United States; 2 University of Texas Health Science Center at Houston School of Biomedical Informatics Houston, TX United States

**Keywords:** symptom, self report, telemedicine, technology, internet, mobile phone, patient preference, cancer, patient-reported outcomes

## Abstract

**Background:**

Electronic symptom self-reporting systems (e-SRS) have been shown to improve symptoms and survival in patients with cancer. However, patient engagement in using e-SRS for voluntary symptom self-reporting is less optimal. Multiple factors can potentially affect patients’ acceptance and engagement in using home-based e-SRS. However, such factors have not been fully explored in cancer populations.

**Objective:**

The aim of this study is to understand the acceptance and use of home-based e-SRS by patients with cancer and identify associated facilitators and barriers.

**Methods:**

PubMed, CINAHL, Scopus, and PsycINFO (January 2010 to March 2020) were searched using a combination of Medical Subject Headings (MeSH) terms and keywords such as symptom self-reporting, electronic/technology, cancer, and their synonyms. Included studies focused on the use of home-based e-SRS by patients with cancer and their families. Studies on patients’ use of e-SRS in clinical settings only were excluded. Of the 3740 papers retrieved, 33 were included in the final review. Factors associated with patient acceptance and use of e-SRS were extracted and synthesized.

**Results:**

Most e-SRS were web based (22/33, 66%) or mobile app based (9/33, 27%). The e-SRS initial acceptance, represented by patient enrollment rates, ranged from 40% (22/55) to 100% (100/100). High e-SRS acceptance was rated by 69% (59/85) to 77.6% (337/434) of the patients after they used the system. The e-SRS use, measured by patients’ response rates to questionnaires (ranging from 1596/3521, 45.33% to 92%) or system log-on rates (ranging from 4/12, 33% to 99/100, 99%), declined over time in general patterns. Few studies (n=7) reported e-SRS use beyond 6 months, with the response rates ranging from 62% (40/64) to 85.1% (541/636) and the log-on rates ranging from 63.6% (103/162) to 77% (49/64). The availability of compatible devices and technical support, interactive system features, information accessibility, privacy, questionnaire quality, patient physical/psychosocial status, and age were associated with patient acceptance and use of home-based e-SRS.

**Conclusions:**

Acceptance and use of home-based e-SRS by patients with cancer varied significantly across studies, as assessed by a variety of approaches. The lack of access to technology has remained a barrier to e-SRS adoption. Interactive system features and personalized questionnaires may increase patient engagement. More studies are needed to further understand patients’ long-term use of home-based e-SRS behavior patterns to develop personalized interventions to support symptom self-management and self-reporting of patients with cancer for optimal health outcomes.

## Introduction

### Background

Patient-reported symptoms, as patient-reported outcomes, are directly reported by patients without any editing or interpretation by clinicians [1,2]. The importance of collecting patient-reported symptoms has been increasingly recognized in cancer care because patients with cancer often experience unpredictable subjective symptoms, such as severe nausea, fatigue, or pain, which can lead to unwarranted emergency room visits or hospital admissions [2-4]. Multiple studies have shown that clinicians are less reliable in identifying subjective symptoms than patients; clinicians are more likely to underestimate the severity of symptoms and sometimes overlook the patient’s self-report [5]. Thus, collecting symptom information directly from patients with cancer is an important component of effective symptom management and improved quality of cancer care.

There is growing evidence for the use of electronic technology systems to collect patient-reported symptoms [5]. Electronic symptom self-reporting systems (e-SRS) have a variety of advantages compared with paper-and-pencil–based reporting formats, including fewer errors in data entry, less missing data, less burden in data management, faster access to data, increased potential for adopting alerts and notifications, and improved real-time patient-provider communications [6-8]. For example, one study found that persons using paper diaries for tracking pain reported a high level of fake compliance (90% of patients reported the use of paper diaries for pain tracking, but only 32% actually used), whereas the electronic diaries group demonstrated 99% validated compliance [9].

Informed by the chronic care model, patients with cancer and their families are expected to be in partnership with clinicians for joint management of the disease and related consequences to improve the quality of cancer care [10]. Remote symptom reporting using electronic technology systems outside cancer clinic settings, that is, using telehealth, play an increasingly significant role in this partnership [11]. Using home-based e-SRS, patients can report their signs and symptoms earlier than waiting for their next clinical visits, facilitating more efficient and effective symptom management [5,12]. Home-based e-SRS can be cost-effective because of the low cost of data collection using electronic surveys and timely identification and management of early symptoms before becoming severe [11,13]. Although many studies have collected patient-reported symptoms in clinical settings [14-16], research has found that patients with cancer usually report fewer and/or less severe symptoms during clinical visits than when self-reported in real time from home [17]. In addition, clinic-based reporting systems may not be optimal for patients receiving oral anticancer therapies, who often have less frequent clinical follow-up visits.

e-SRS has been shown to improve symptoms and survival in patients with cancer [12,18,19]. However, to achieve these benefits, patients’ acceptance and voluntary use of home-based e-SRS are essential for establishing long-term benefits of symptom self-reporting [8,20]. A literature review of 33 e-SRS used in cancer care highlights that 70% of reporting systems were provided with in-clinic access [18]. To date, there has been no synthesized evaluation of what is known about voluntary use of home-based e-SRS by patients with cancer. Multiple personal and technical factors can potentially affect patients’ acceptance and use of home-based e-SRS; however, such factors have not been fully explored among cancer populations.

### Objectives

This study aims to explore acceptance and use of home-based e-SRS by patients with cancer and facilitators/barriers associated with acceptance and use of home-based e-SRS by patients with cancer.

## Methods

### Search Strategy

Databases, including PubMed, CINAHL, Scopus, and PsycINFO, were searched for papers published between January 2010 and March 2020. A total of 3 groups of search terms—symptom self-reporting, electronic/technology, and cancer/oncology—were used in combination with their Medical Subject Headings (MeSH) terms, keywords, and synonyms. Synonyms were generated based on preliminary searches and some entry terms of MeSH terms (search strategies are included in Multimedia Appendix 1). We included papers that (1) included patients diagnosed with cancer who were aged ≥18 years, (2) reported patients or family members’ use of an electronic version of symptom self-reporting systems/tools for symptom self-reporting outside of clinic or hospital settings, and (3) were original peer-reviewed research papers that were written in English. Studies published before 2010 were excluded because smartphones and tablets were not widely used until 2010. We excluded papers that reported the use of paper-based symptom self-reporting tools or clinic-based e-SRS only. Other excluded papers were those that did not provide measures or results specifically about patients’ acceptance or use of home-based e-SRS or did not focus on symptom reporting.

### Selection of Papers

**Figure 1:**
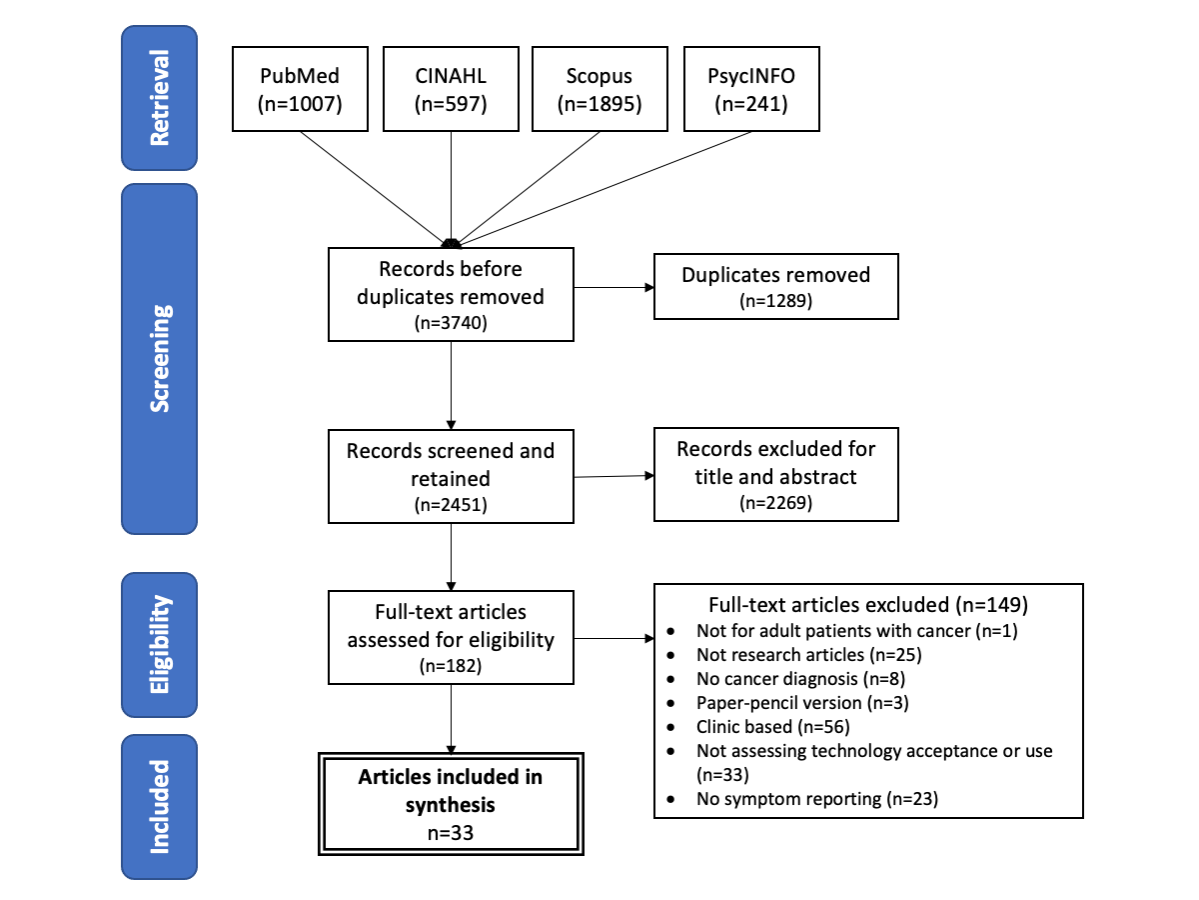
PRISMA (Preferred Reporting Items for Systematic Reviews and Meta-Analyses) chart.

A total of 3740 papers were retrieved from database searches. After removing duplicates and reviewing titles and abstracts for relevance, 182 papers remained for the full-text screening. Among them, 149 papers did not meet the inclusion criteria and were excluded, including not for adults (n=1), no cancer diagnosis (n=8), not research papers (n=25), used paper-pencil version of symptom reporting (n=3), not assessing acceptance and use (n=33), not symptom reporting (n=23), and clinic-based systems (n=56). A total of 33 papers were included in the final review. Figure 1 shows the PRISMA (Preferred Reporting Items for Systematic Reviews and Meta-Analyses) flowchart describing the overall search and selection process.

### Data Extraction and Analysis

Study characteristics and information regarding e-SRS were extracted from each reviewed paper. Information regarding e-SRS acceptance and use was extracted from user surveys and postintervention interviews. Technology *acceptance* and *use* were defined based on the widely adopted Unified Theory of Acceptance and Use of Technology (UTAUT) [21]. Specifically, in this study, e-SRS acceptance was defined as patients’ intention to use home-based e-SRS. Among the studies that did not directly assess patients’ intention to use e-SRS before their actual use of the system, e-SRS initial acceptance was operationalized as patients’ willingness to participate in the study using home-based e-SRS (eg, participant enrollment rate: the rate of enrollees out of all approached eligible patients) [22]. The e-SRS use was defined as the actual use rate of e-SRS or the description of patients’ e-SRS use behavior. The e-SRS use rates were extracted and summarized based on the calculations reported in the studies, categorized as long-term (≥6 months) and short-term (<6 months) use. Potential facilitators/barriers to e-SRS acceptance and use were extracted and synthesized based on reported reasons for nonparticipation, users’ feedback surveys, and postintervention interviews.

### Critical Appraisal for Quality of Studies

The quality of studies was assessed using the Methodological Index for Nonrandomized Studies (MINORS), which includes 8 items for assessing noncomparative nonrandomized studies and 4 optional items for comparative studies (global Cronbach α=.73) [23]. As this study focused on users’ acceptance and use of e-SRS, if a comparative study was reported, only the information from the technology user groups were analyzed. Therefore, this study only adopted the first 8 criteria of MINORS, including whether the study has a clear aim, clear inclusion and exclusion criteria, prospective data collection, appropriate endpoints, unbiased assessment, adequate study period, reasonable proportion of follow-up loss, and prospective sample size calculation [23]. Furthermore, the Critical Appraisal Skills Programme (CASP) Qualitative Research Checklist was used to evaluate the quality of a qualitative study or the qualitative design of a mixed methods study [24]. This 10-item checklist assesses the appropriateness of qualitative methodology, design, data collection, and analysis process, and the value of findings [24]. Items from both the MINORS and CASP checklist were graded on a scale of 0 (not reported), 1 (reported but inadequate), and 2 (reported and adequate). Studies with a MINORS score of 11 (out of a total score of 16) or less, or a CASP checklist score less than 15 (out of 20) were classified as low-quality studies [25,26]. Overall, both MINORS and CASP scores indicated adequate quality of the reviewed studies. The mean MINORS score was 13.6 (SD 1.4), with a range of 10-16 out of 16. Only 2 studies had a low-quality score below 11, mainly due to small sample sizes (n=5-21) and inadequate description of the study endpoints [27,28]. The mean CASP checklist score was 17.7 (SD 1.5) out of 20 (range, 15-20). No study had a low-quality score below 15 (Multimedia Appendix 2).

## Results

### Summary of Study Characteristics

Among the reviewed papers, most studies (1) were conducted in the United States (15/33, 45%) or Europe (16/33, 48%), (2) recruited participants from tertiary cancer centers (25/33, 76%), (3) had sample sizes ranging from 5 to 3521, and (4) reported a sample size smaller than 50 (18/33, 55%). Among the total of 7382 participants in all studies, the majority were patients diagnosed with breast cancer (1771/7382, 23.99%; Multimedia Appendix 3). A total of 27 studies (27/33, 82%) targeted patients on active anticancer treatment, and 13 studies (13/33, 39%) targeted patients with chemotherapy/endocrine therapy/immunotherapy (Tables 1 and 2). The majority of the studies had a quasi-experimental study design (25/33, 76%). The remaining studies used experimental (7/33, 21%), mixed methods (7/33, 21%), case control (1/33, 3%), and qualitative designs (1/33, 3%).

**Table 1:** Anticancer treatment type (N=33 studies).

Anticancer treatment types	Studies, n (%)
Surgery	6 (18)
Chemotherapy or hormonotherapy or immunotherapy	13 (39)
Radiation	7 (21)
All types mentioned	2 (6)
Unknown	5 (15)

**Table 2:** Anticancer treatment status (N=33 studies).

Anticancer treatment status	Studies, n (%)
On active treatment	27 (82)
Either on active treatment or survivors after treatment	2 (6)
Unknown	4 (12)

In addition to assessing patients’ acceptance and use of home-based e-SRS in most studies, 1 study also reported caregivers’ attitudes and preferences toward e-SRS [29]. One study reported that 8 out of 92 participants actually had their family caregivers who reported their symptoms for them, whereas there was no information provided regarding caregivers’ acceptance and use in this study [30]. Study durations ranged from 1 month to 24 months, of which 7 studies (7/33, 21%) [31-37] followed up with participants for more than 6 months (Multimedia Appendix 2).

In total, 17 web-based e-SRS were reported in 22 studies (22/33, 66%) [27-29,31-49], including 2 studies that integrated web-based platforms with patient portals and electronic health records (EHRs) [41,47]. A total of 9 studies presented 7 mobile app–based e-SRS (9/33, 27%) [30,50-57], 1 study reported an interactive voice response system [58], and 1 study used only text messaging for symptom reporting [59]. The most commonly adopted symptom reporting instruments or questionnaires in home-based e-SRS were the National Cancer Institute-Common Terminology Criteria for Adverse Events or patient-reported outcome version of Common Terminology Criteria for Adverse Events (6/33, 19%) [27,28,30,38,46,48] and the European Organisation for Research and Treatment of Cancer Questionnaires (4/33, 12%) [28,31,45,49]. A total of 5 studies (5/33, 15%) required patients to report at least one of the several listed symptoms [40,42,54,57,58]. Most studies specified a reporting frequency, including daily (n=11), weekly (n=10), every other week (n=2), monthly (n=1), and less frequent than monthly (n=3), although a small number allowed patients to report their symptoms whenever they wanted (4/33, 12%) and 2 studies (2/33, 6%) did not report reporting frequencies (Multimedia Appendix 2).

### e-SRS Acceptance

None of the 33 studies assessed patients’ initial intention to use e-SRS before actual use. A total of 23 studies quantified patients’ enrollment rates in e-SRS studies and reported a median rate of 68% (range, 22/25-18/18, 40%-100%) [27-30,32-34,39-42,44,46-49,51-53,55,56,58,59]. Mobile app–based systems showed lower enrollment rates than that of web-based systems (median 57% vs 71%). Among the 7 out of 23 studies that used mobile app–based systems [30,50-53,55,56], 4 studies (enrollment rates=40%-57%, 22/25-38/67) [50,51,53,56] showed that the most common reason for rejection was that patients did not have devices (eg, smartphones) or their devices were not compatible with the e-SRS platform (eg, iPhone or Android phone mismatched). A total of 2 studies using mobile app–based systems had relatively high enrollment rates (64/75, 85% and 66/107, 61.7%) because both studies provided mobile devices for participation [52,55].

A total of 7 studies assessed patients’ technology acceptance after they used the systems [31,39-41,49,50,52]. Four of them reported that over 75% (56/75) of the patients stated, “I would continue to use it if asked.” [31,39,40,52]. In addition, 5 studies reported that over 69% (59/85) stated, “I would recommend it to others.” [40,41,49,50,52]. One study reported that 80% (337/434) of the patients preferred e-SRS to the paper-and-pencil–based format in the future [31].

### e-SRS Use

Patients’ use of e-SRS was measured in various ways across the studies (Multimedia Appendix 4); one of the most common methods was to assess questionnaire response rates. However, the calculation of the response rate varied among studies. Several studies calculated the response rate using the number of patients who had ever reported their symptoms during certain time frames divided by the total number of all enrolled patients [29-32,36,40,42-44,46,48,50]. Overall, the mean percentage of the participants who had ever used e-SRS ranged from 70% (442/631) to 92% (45/49) across studies. A few other studies calculated the response rate using the number of submitted symptoms/forms divided by the total number of all expected forms. Using this method, the overall mean response rates across studies ranged from 45% (1596/3521) to 90% [29,35,37-39,42,49,52,53,55,56,58,59].

The log-on rate, that is, the frequency of accessing the system, was also adopted in some studies to measure the e-SRS use [30,33,39,45,46,53]. The log-on rate was calculated as the number of patients who logged on to systems divided by the number of all enrolled patients, which ranged from 33% (4/12) to 99% (99/100) across studies [30,34,45,46]. Some studies reported the average number of log-ons or the number of log-on days during the entire study period. For example, 1 study reported an average of 4 patient log-ons during a 30-day study period [42], another study reported an average of 17 log-ons over 34 weeks [36], and an average of 22 log-on days was reported during an average follow-up period of 12.70 months [53]. However, no study has reported the relationship between the log-on rates and the rates of actual symptom reporting.

Among the studies that assessed the change in e-SRS use over time [35,36,42,47,56], 2 longitudinal use patterns were identified. One pattern was the increased use from the beginning to nearly the midpoint of the study period (eg, initial 2 weeks of a 4-week study, 11-14 weeks of a 24-week study, or 16 weeks of a 34-week study), followed by a gradual decrease in use until the end of the study [35,36,42]. The second pattern was that the e-SRS use decreased over time throughout the study period [31,47,56]. For example, 85.1% (541/636) of patients used e-SRS within the first 6 months of one study, whereas the percentage decreased to 70% (442/631) at 9 months and 66.3% (414/624) at 15 months [31]. Overall, for the long-term use of e-SRS, the response rates ranged from 62% (40/64) to 85.1% (541/636) [31,33,35-37] and the log-on rates were 63.6% (103/162) to 77% (49/64) [33,44].

### Facilitators/Barriers Associated With Home-Based e-SRS Acceptance and Use

#### Technology-Related Factors

The most commonly reported reasons for patients’ reluctance to participate in e-SRS studies were the lack of access to compatible devices (eg, computers, smartphones, or tablets) [31,46,50,51,53,58]; lack of access to the internet [31,46]; or limited experience with computers, smartphones, or the internet [33,37,53,59]. A few studies excluded patients who did not have access to compatible devices or who did not have active email/patient portal accounts [36,39,40,42,43,51]. Only 1 study provided desktop computers [44], and 3 studies provided mobile devices to participants [52,55,57]. Patients with more technology experience had fewer technical issues and higher use of e-SRS [32,33,37,48,49]. Some patients could not use the systems owing to the failure of downloading apps [50] or incompatible operating systems with their devices [56].

#### e-SRS Features

Multiple studies reported patients’ preferences for interactive system features, such as automatic reminders for symptom self-reporting [27,31,42,43,46,55,56] and health care providers’ follow-up with self-reported symptoms [29,38,42,43,45,54,55]. System-generated self-management recommendations contributed to patients’ high use and satisfaction [29,38,46,49,54,55]. Patients also favored the systems’ features of (1) tracking symptoms over time [54,55], (2) bookmarking [31,34], (3) summarizing the symptom review [31], (4) having an icon- and image-based interface [30], (5) interacting with other patients [32], (6) reporting in free-text format [34], (7) connecting to EHR [41], and (8) interoperating with mobile devices [41].

#### Symptom Reporting Questionnaires

The quality of symptom questionnaires potentially affected patients’ acceptance and use of e-SRS for symptom self-reporting [29,31,38,52,54,55]. Some patients complained about the overload or overlap of questions in the questionnaires [31,52,54] or questions that were difficult to understand [29,38,55]. Patients sometimes lost interest in using the system because the symptoms listed in the questionnaires were irrelevant to the symptoms they wanted to report, and the simple grading scale that requires patients to grade the presence or severity of certain symptoms was confusing [31,52,54].

#### Physical Health and Psychosocial Status

Health status of patients was associated with their acceptance and use of e-SRS. Patients often missed reporting of symptoms because of their illness, and about 7% of the missing data were due to the patients who were too ill to complete the questionnaires [36]. Some patients expressed their dislike or disinterest in participating in e-SRS studies because they felt that they were too tired (lack of energy) to engage in routine electronic symptom self-reporting [36,45,50,53,56,58,59]. Patients with brain tumors, such as glioma, struggled to use the technology because of the loss of their hand strength and poor memory [29]. Visual impairments in older people disrupted the use of electronic systems [34].

Qualitative interviews showed that patients’ level of self-confidence and control in managing their health played an important role in their use of e-SRS [29,55], whereas some studies reported that increased symptom-related stress was associated with increased e-SRS use [32,34,42]. Some other studies indicated that patients might worry about the increased awareness of their symptoms through symptom tracking and reporting [33,46,52,53]. One study reported that patients were reluctant to use the system because they were afraid of being overly focused on their unpleasant symptoms, and the constant detection of minor symptoms that they usually ignored might eventually make them mentally exhausted [29].

#### Home-Based Reporting

Patients were satisfied with their use of e-SRS at home because of (1) the convenience of flexible times and frequencies of reporting [29,31,34,43]; (2) timely symptom reporting, particularly for acute symptoms [31,43]; (3) benefits for patients who had concerns about language barriers [29] and who lived far from clinics [42]; and (4) reduction of clinic visit durations as clinicians have already been aware of their symptoms [39]. However, some patients might have concerns about the lack of face-to-face interactions with their providers by using e-SRS [29,38].

#### Demographic Factors

Patients who enrolled in the e-SRS studies had a mean age of 54 to 64 years, and patients who did not enroll had a mean age of 62.2 to 66 years [31,45,50,59]. Younger age [31,34,45,48,50,59], higher education level [36,38,48,49], White race [36,41,45], and male sex [31,36] were associated with higher acceptance and use of e-SRS. Evidence regarding the influence of employment status [49,56] and cancer staging [36,41] was mixed.

## Discussion

### Principal Findings

To the best of our knowledge, this study is the first to focus on acceptance and use of home-based e-SRS for symptom self-reporting by patients with cancer. This study also explored potential facilitators and barriers to e-SRS acceptance and use. Home-based e-SRS demonstrated the advantages of convenience, flexibility, and on-time symptom reporting. In addition, it enhanced patients’ self-confidence in symptom control during/after their cancer treatment. However, considering the various participation rates and diverse reasons for nonuse of the systems reported, this study identified that the lack of technology compatibility was still a significant barrier to patients’ adoption of home-based e-SRS. Although providing eHealth services or mobile devices to patients may help meet their needs for technology access, from the system design and development perspective, increasing the compatibility of e-SRS on multiple platforms seems to be a more potentially effective solution. Furthermore, system features, quality of symptom questionnaires, characteristics and health status of patients, and perceived benefits of using the systems were the important factors associated with acceptance and use of home-based e-SRS by patients with cancer. These findings are in line with literature reports that patient engagement in digital health interventions is associated with personal agency, motivation, and the quality of digital health interventions [22].

The review revealed that inconsistent approaches were used to assess e-SRS acceptance or use across studies, which might be because of different study purposes and designs. For example, the purpose of quasi-experimental pilot or feasibility studies was to investigate the feasibility of recruiting participants in e-SRS studies, in which enrollment rates served as an indirect assessment of patient acceptance of e-SRS [31]. Randomized controlled trials had aimed to evaluate the effects of the technology interventions on patient outcomes, in which participants’ exposure to the technology systems was usually defined by a minimal threshold of access to the system or assessed based on patient outcomes of interest [49]. Inconsistent measurement and reporting of e-SRS acceptance and use across studies made the synthesis of findings challenging [19]. According to the widely adopted technology acceptance model, technology acceptance can be directly assessed by the person’s statements regarding her/his behavioral intention to the use of technology [60]. The assessment of the actual use of technology is complex, as the expected outcomes of technology use vary by systems. Although log files are commonly used to measure how frequently users use technology systems, the log-in to the systems does not always accurately reflect users’ actual use of the systems for expected outcomes. In the case of use of home-based e-SRS, users may log into the system, but they do not use the reporting function to report their symptoms. Overall, the measure of the actual use of technology should consider whether the users have performed the technology functionalities for expected outcomes.

Among all identified facilitators of and barriers to e-SRS acceptance and use, interactive features of e-SRS and the quality of symptom reporting questionnaires are considered as modifiable factors that can be purposefully modified and upgraded to meet users’ needs, compared with nonmodifiable factors such as patients’ demographic or clinical characteristics. In general, patients preferred regular reminders for their use of e-SRS and the feature of receiving feedback from either automated self-management advice or their health care team on their reported symptoms, which can be considered essential features in home-based e-SRS [19]. Although patients favored the integration of electronic symptom reporting with their electronic medical records, not many current home-based e-SRS have considered interoperability with clinical information systems [41,47]. Such limitations in system design and development should be addressed in future upgrades. In addition, a well-designed personalized questionnaire or a personalized way to deliver the questionnaire, for example, using a specific symptom-focused questionnaire or a specific type of treatment-focused questionnaire seems to encourage patients’ use of e-SRS for voluntary symptom self-reporting.

This study suggested that clinicians’ feedback on patients’ symptom reporting through e-SRS was a facilitator of patient use of the system. The literature indicates that patients’ use of e-SRS for symptom self-reporting provides opportunities for clinicians to understand patients’ dynamic needs over time and facilitates productive interactions and interpersonal relationships between patients and clinicians [10,61]. Within the current outpatient oncology model, particularly with the increasing use of oral anticancer treatment at home, patients with cancer have fewer opportunities to directly interact with their health care professionals during office visits, share the concerns of their symptoms experienced, or receive sufficient information for symptom self-management [62]. The paradigm shift in cancer care delivery encourages the adoption of novel platforms for more effective patient-provider communication to support patient-centered care. Although the emerging home-based e-SRS provides the opportunity to engage and empower patients and families in health communication and symptom self-management, the adoption of home-based e-SRS from the health system and health professional side remains unclear [21]. None of the reviewed studies reported clinicians’ interactions with home-based e-SRS, that is, clinicians’ acceptance and use of symptom information reported from the system. To fill this gap, the design and development of home-based e-SRS should consider providers’ preferences for the way to interact with home-based e-SRS and health systems’ expectations for the integration of home-based e-SRS into clinical workflows, to close the loop for optimal care delivery.

This study identified a minimal number of studies evaluating long-term e-SRS use (≥6 months) [31-37]. Despite the increasing recognition of the importance of patient symptom self-reporting throughout the cancer care trajectory, patients’ long-term use of home-based e-SRS for symptom self-reporting tended to decline over time. The potential dynamic e-SRS use patterns identified in this study suggest that more studies are needed to increase our understanding of patients’ long-term e-SRS use behaviors. Further exploration of factors associated with e-SRS long-term use trajectory patterns will contribute to the development of personalized support for patients’ use of e-SRS for symptom self-reporting.

Patients’ expectations and motivations for interacting with reporting systems may also change over time. For example, expectations for using e-SRS vary with patient health status. Interestingly, patients’ health status could be either positively or negatively associated with their technology use behavior [20,63]. Patients with increased symptom distress might be motivated to continue tracking and reporting their symptoms to health care professionals. However, it was also possible that some patients decreased their use of e-SRS because they wanted to ignore the deterioration of their health status [36,46]. Further studies can explore the conditions and contexts that interfere with patients’ use of e-SRS when their health status improves or declines.

Patients’ feedback from postintervention interviews and surveys revealed that patients were more likely to continue using e-SRS after realizing that the systems were useful and convenient to use. Despite the low level of e-SRS acceptance (ie, low enrollment rates) before using the systems, patients’ behavioral intention to use e-SRS improved after their actual use. We did not identify any study that provided patients with information or training before using a home-based e-SRS. Therefore, it is unknown whether patients’ behavioral intentions, including perceived usefulness and ease of use, change after exposure to e-SRS. In future studies, it would be interesting to investigate how proactive and individualized training sessions reinforce patients’ use of e-SRS for symptom self-reporting.

According to the UTAUT model, age, gender, and previous technology experiences potentially moderate the effects of determinants on the actual behavior of technology use [21]. Consistent with the UTAUT model, these personal factors were also identified in this study, especially age and previous technology experiences. These factors are not modifiable but may contribute to the development of targeted interventions and support for specific subgroups of the population. It is well known that family caregivers are commonly involved in medication and symptom self-management of patients with cancer, particularly for older adults with cancer [64]. This study indicated that family caregivers were sometimes the persons who actually used home-based e-SRS to report symptoms for their family members. It is also important to understand family caregivers’ opinions of e-SRS use, as family caregivers’ perceptions of symptom distress and symptom reporting may not always be congruent with those of patients with cancer [65]. However, there was a lack of research on cancer patient family caregivers’ acceptance and use of e-SRS, which can be another important research topic in the future.

Patients appreciated that they had fewer time constraints in home-based reporting, and they reported their symptoms in real time without concerns of recalling their symptom experiences during their clinic visits. Furthermore, remote home-based symptom self-reporting could be especially beneficial for the underserved population who have geographic barriers to access health care or language problems for health care communications [29,42]. Of note, at the time of this study, the COVID-19 pandemic was pushing patients and clinicians to find new ways to work together. Perhaps now, more than ever, is the time to encourage patient adoption of e-SRS in cancer care in ways that can efficiently inform the conversation between clinicians and patients during a virtual telehealth clinical visit. The results of this study provide insight into how to engage patients in the use of e-SRS to facilitate telehealth care to improve health outcomes.

### Limitations

This study has several limitations. First, many studies had small sample sizes and did not include diverse populations. Thus, there was a risk of selection bias. Second, the literature search was limited to studies published from January 2010 to March 2020 and to English language papers. Studies published before 2010 and studies published in non-English languages may contain useful information regarding patients’ opinions on using e-SRS at home. Finally, this study considered participant enrollment rates in e-SRS studies as a surrogate measure of acceptance of e-SRS by patients with cancer. Such indirect measures might be less accurate, as some patients might refuse e-SRS studies for reasons that were not related to their acceptance to use e-SRS for symptom reporting. We focused on studies that reported the reasons for nonparticipation and extracted those that were potentially relevant to technology acceptance.

### Conclusions

There is a growing interest in managing symptoms of patients with cancer remotely over time using electronic technology systems. Home-based e-SRS provides opportunities for patients with cancer to engage in symptom self-reporting from the initial cancer diagnosis, throughout treatment, and well into survivorship. It is important to evaluate patients’ acceptance and use of e-SRS with standardized assessments so that the sustainability of the systems will be possible. Furthermore, understanding the facilitators and barriers of e-SRS regarding its acceptance and use in home settings will enhance the dissemination of e-SRS in routine cancer care by patients with cancer. This study highlights the importance of assessing patients’ accessibility to technology, physical and psychosocial status, and demographic factors for optimal symptom self-reporting. In addition, the design and development of interactive system features and personalized symptom reporting questionnaires should be considered to increase patient engagement. Future studies should explore long-term e-SRS use behavioral patterns of patients and develop personalized interventions to support symptom self-management and self-reporting for optimal health-related outcomes of patients with cancer.

## Appendix

Multimedia Appendix 1 Search strategies.

Multimedia Appendix 2 Study characteristics.

Multimedia Appendix 3 The number of participants by cancer type (n=7382 participants).

Multimedia Appendix 4 Measures of patient use of electronic symptom self-reporting system in the studies.

## Abbreviations

CASP: Critical Appraisal Skills Programme
EHR: electronic health record
e-SRS: electronic symptom self-reporting system
MeSH: Medical Subject Headings
MINORS: Methodological Index for Nonrandomized Studies
UTAUT: Unified Theory of Acceptance and Use of Technology
