# Supplementary material for: Acceptance and Use of Home-Based Electronic Symptom Self-Reporting Systems in Patients With Cancer: Systematic Review
Source: J Med Internet Res. 2021 Mar 12;23(3):e24638. doi: 10.2196/24638 (PMC7998328; doi:10.2196/24638)
Supplement: Multimedia Appendix 1 [file jmir_v23i3e24638_app1.doc]

Multimedia Appendix I:

Table 1. Search Strategies

|  | “Self-Reporting” | “Electronic/Technology” | “Cancer/Oncology” |
| --- | --- | --- | --- |
| PubMed | ("Self Report"[Mesh] OR “Self-Assessment"[Mesh] OR "Patient Reported Outcome Measures"[Mesh] OR "patient reported outcome"[tiab] OR "patient reported outcomes"[tiab] OR "patient-reported outcome"[tiab] OR "patient-reported outcomes"[tiab] OR PROM[tiab] OR “self-report”[tiab] OR "self-reporting"[tiab] OR "self-reported"[tiab] OR “self-assess”[tiab] OR “self-assessment”[tiab] OR “symptom report”[tiab] OR "symptom reporting"[tiab] OR "symptom monitor"[tiab] OR "symptom monitoring"[tiab] | "Electronic Mail"[Mesh] OR "Telemedicine"[Mesh] OR "Computers"[Mesh] OR "Computers, Handheld"[Mesh] OR "Mobile Applications"[Mesh] OR "Cell Phone"[Mesh] OR "Smartphone"[Mesh] OR electronic[tiab] OR ePRO[tiab] OR tablet[tiab] OR tablets[tiab] OR computer[tiab] OR computers[tiab] OR smartphone[tiab] OR smartphones[tiab] OR mobile[tiab] OR mobiles[tiab] | "Neoplasms"[Mesh] OR "Medical Oncology"[Mesh] OR neoplasm[tiab] OR neoplasms[tiab] OR neoplasia[tiab] OR neoplasias[tiab] OR malignancy[tiab] OR malignant[tiab] OR malignancies[tiab] OR cancer[tiab] OR cancers[tiab] OR tumor[tiab] OR tumors[tiab] OR oncology[tiab] OR oncologic[tiab] |
| CINAHL | (MH "Self Report") OR (MH "Self Assessment") OR (MH "Patient-Reported Outcomes") OR TI("patient reported outcome" OR "patient reported outcomes" OR "patient-reported outcome" OR "patient-reported outcomes" OR PROM OR “self-report” OR "self-reporting" OR "self-reported" OR “self-assess” OR “self-assessment” OR “symptom report” OR "symptom reporting" OR "symptom monitor" OR "symptom monitoring") OR AB("patient reported outcome" OR "patient reported outcomes" OR "patient-reported outcome" OR "patient-reported outcomes" OR PROM OR “self-report” OR "self-reporting" OR "self-reported" OR “symptom report” OR "symptom reporting" OR "symptom monitor" OR "symptom monitoring") | (MH "Email") OR (MH "Telehealth") OR (MH "Telemedicine") OR (MH "Smartphone") OR (MH "Mobile Applications") OR (MH "Computers, Hand-Held") OR (MH "Cellular Phone") OR TI(electronic OR ePRO OR tablet OR tablets OR computer OR computers OR smartphone OR smartphones OR mobile OR mobiles) OR AB(electronic OR ePRO OR tablet OR tablets OR computer OR computers OR smartphone OR smartphones OR mobile OR mobiles) | (MH "Neoplasms") OR (MH "Cancer Patients") OR (MH "Cancer Survivors") OR (MH "Oncology") OR (MH "Oncologic Care") OR TI(neoplasm OR neoplasms OR neoplasia OR neoplasias OR malignancy OR malignant OR malignancies OR cancer OR cancers OR tumor OR tumors OR oncology OR oncologic) OR AB(neoplasm OR neoplasms OR neoplasia OR neoplasias OR malignancy OR malignant OR malignancies OR cancer OR cancers OR tumor OR tumors OR oncology OR oncologic) |
| Scopus | TITLE-ABS-KEY ("patient reported" OR "patient-reported" OR "patient reported outcome" OR "patient-reported outcome" OR "patient reported outcomes" OR "patient-reported outcomes" OR "self report" OR "self-report" OR "self assess" OR "self-assess" OR "self assessment" OR "self-assessment" OR "symptom report" OR "symptom reporting" OR "symptom assess" OR "symptom assessment") | TITLE-ABS-KEY ("electronic mail" OR "email" OR "computer" OR "computers" OR "handheld computer" OR "handheld computers" OR "mobile" OR "mobiles" OR "mobile application" OR "mobile applications" OR "cell phone" OR "smartphone" OR "tablet" OR "tablets" OR "electronic" OR "ePRO" OR "telemedicine") | TITLE-ABS-KEY ("cancer" OR "malignancy" OR "oncology" OR “tumor” OR “neoplasm”) |
| PsycINFO | (DE "Self-Analysis" OR DE "Self-Evaluation" OR DE "Self-Monitoring" OR DE "Self-Report" OR DE “Patient Reported Outcome Measures”) OR TI("patient reported outcome" OR "patient reported outcomes" OR "patient-reported outcome" OR "patient-reported outcomes" OR PROM OR “self-report” OR "self-reporting" OR "self-reported" OR “self-assess” OR “self-assessment” OR “symptom report” OR "symptom reporting" OR "symptom monitor" OR "symptom monitoring") OR AB("patient reported outcome" OR "patient reported outcomes" OR "patient-reported outcome" OR "patient-reported outcomes" OR PROM OR “self-report” OR "self-reporting" OR "self-reported" OR “symptom report” OR "symptom reporting" OR "symptom monitor" OR "symptom monitoring") | (DE "Computer Mediated Communication" OR DE "Electronic Communication" OR DE "Computer Applications" OR DE "Information and Communication Technology" OR DE "Internet" OR DE "Mobile Devices" OR DE "Telemedicine" OR DE "Smartphone Use" OR DE "Smartphones" OR DE "Tablet Computers" OR DE "Mobile Applications" DE "Mobile Phones" OR DE "Mobile Technology") OR TI(electronic OR ePRO OR tablet OR tablets OR computer OR computers OR smartphone OR smartphones OR mobile OR mobiles) OR AB(electronic OR ePRO OR tablet OR tablets OR computer OR computers OR smartphone OR smartphones OR mobile OR mobiles) | (DE "Neoplasms" OR DE "Oncology") OR TI(neoplasm OR neoplasms OR neoplasia OR neoplasias OR malignancy OR malignant OR malignancies OR cancer OR cancers OR tumor OR tumors OR oncology OR oncologic) OR AB(neoplasm OR neoplasms OR neoplasia OR neoplasias OR malignancy OR malignant OR malignancies OR cancer OR cancers OR tumor OR tumors OR oncology OR oncologic) |
