# Supplementary material for: Acceptance and Use of Home-Based Electronic Symptom Self-Reporting Systems in Patients With Cancer: Systematic Review
Source: J Med Internet Res. 2021 Mar 12;23(3):e24638. doi: 10.2196/24638 (PMC7998328; doi:10.2196/24638)
Supplement: Multimedia Appendix 2 [file jmir_v23i3e24638_app2.doc]

Multimedia Appendix II:

Table 2. Study Characteristics

| **Author (Year)** | **Country** | **Setting** | **Design** | **Platform** | **Name of the System** | **Type of Cancer & Treatment** | **Sample** | **Instruments for symptom measurement** | **Reporting timepoints & Study duration** | **MINORS** | **CASP** |
| --- | --- | --- | --- | --- | --- | --- | --- | --- | --- | --- | --- |
| Andikyan et al. (2012) [48] | USA | tertiary cancer center | Quasi-experimental pilot study | Web-based | STAR (Symptom Tracking and Reporting for Patients) | Gynecologic cancer  Surgery | n=49  Median age 56 (range 23-74)  16% (8 subjects) were ≥65 years old | CTCAE  EORTC QLQ-30 | Pre-operative baseline and weekly  6 weeks | 15 | - |
| Ashley et al. (2013) [31] | UK | tertiary cancer center | Quasi-experimental study  Feasibility test | Web-based | ePOCS (Electronic Patient-reported Outcomes from Cancer Survivors) | Breast, colorectal, prostate cancer  Unknown | n=636  Men (43.1%) median age 66 (range, 23-92)  Women (56.9%) median age 58 (range, 24-88) | EORTC QLQ BR23, CR29, PR25 (depend on the type of cancer)  IPQ-R: 38-item on 1-5 scale + 14 symptoms on Y/N  EuroQol-5D ver.2: 6-item on a 0-100 scale  SF-36v2: 36-item  SDI-21: 21-item on a 0-3 scale  QLACS: 47 items | T1 (6 months after diagnosis), T2 (9 months after diagnosis), T3 (15 months after diagnosis) | 15 | - |
| Bae et al. (2018) [30] | South Korea | Tertiary cancer center | Quasi-experimental pilot study  Feasibility study | Mobile-based | PRO-SMART | Any type of cancer  Chemotherapy | n=100  Men 66%, Women 34%  Median age 57 (range, 30-76) | CTCAE ver. 4.0: 9 symptoms on a 3-point scale | Whenever desired  Average ﻿44.5 ± 38.8 days | 13 | - |
| Belarimo et al. (2018) [50] | USA | Tertiary cancer center | Quasi-experimental study  Feasibility test | Mobile-based | No name 1  (iPhone only) | Prostate cancer  Surgery | n=20  Mean age 63.53 (range, 47-75) | EPIC-CP  A numeric scale pain questionnaire | Pre-operative baseline EPIC-CP  POD#2: pain assessment  Weekly after catheter removal: EPIC-CP  60 days | 13 | - |
| Berry et al. (2015) [49] | USA | Tertiary cancer center | RCT | Web-based | ESRA-C (Electronic Self-Report Assessment for Cancer) | Any type of cancer  Chemotherapy | n=752  control (n=378), intervention (n=374)  Control group: usual education  Intervention group: usual education + symptom self-reporting using ESRA-C  Intervention group age:  ≥50 years old: n=248  ≤50 years old: n=126 | Symptom Distress Scale (SDS-15)  EORTC-CIPN20  PHQ-9  A 0-10 pain intensity numerical scale  Skin problems questionnaire | Baseline & 3 other timepoints during the treatment | 14 | - |
| Boele et al. (2016) [29] | Netherlands | tertiary cancer center, outpatient clinic | Quasi-experimental study  Qualitative study | Web-based | Oncokompas | Glioma  Unknown | n=18  Age range 28-76 | Patient Concerns Inventory (PCI) | Unknown | - | 18 |
| Borosound et al. (2013) [32] | Norway | Recruited through advertisements in newspapers, on the Norwegian Cancer Society's website | RCT | Web-based | WebChoice | Breast and prostate cancer  Unknown | n=325  intervention (n=162)  Women with breast cancer: 59.3% (n=96)  Men with prostate cancer: 40.7% (n=66)  Median age 57 (range, 35-80) | Memorial Symptom Assessment Scale-Short Form (MSAS-SF): 32 items, 5-point Likert scales  MOS-SS: 20-item  CES-D: 20-item  Cancer Behavior Inventory ver 2.0 for self-efficacy: 33-item  15D preference-based single index for OoL: 15-item | Whenever desired  1 year | 13 | - |
| Borosound et al. (2014) [33] | Norway | tertiary cancer center | RCT | Web-based | IPPC (Internet-based Patient Provider Communication service)  WebChoice | Breast cancer  Surgery, chemotherapy, radiotherapy, or hormonotherapy | n=167  Control group: usual care  Intervention:   - IPPC (Internet-based patient provider communication services) - WebChoice + IPPC   WebChoice (n=64): median age 51 (range 37-79)  IPPC (n=45): median age 50 (range 31-66)  Control group (n=58): median age 53 (range 36-69) | MSAS-SF: 32 items, 5-point Likert scales  SCQ-19  HADS: 14-item  Cancer Behavior Inventory ver. 2.0 for self-efficacy: 33-item | Whenever desired  6 months | 14 | - |
| Brochmman et al. (2016) [34] | Denmark | Tertiary cancer center, outpatient clinic | Quasi-experimental study  Feasibility study | Web-based | No name 2 | Myeloproliferative neoplasms  Unknown | n=118  Mean age 62 (SD 12)  Focus group interview: n=17  - age≥60: n=8, mean age 72 (SD 9)  - age<60: n=9, mean age 48 (SD 9) | MPN-SAF (Myeloproliferative Neoplasm Symptom Assessment Form)  MPN-SAF TSS (Total Symptom Score)  EORTC QLQ-C30 ver. 3.0  BFI (Brief Fatigue Inventory)  SF-36 ver. 2.0 | Providers could select the different schedule: ﻿once, once a week, once a month and every 3 months | 13 | 18 |
| Cowan et al. (2016) [38] | USA | tertiary cancer center | Quasi-experimental study  Feasibility study  *Update of Andikyan et al. (2012)'s pilot study | Web-based | STAR | Gynecologic cancer  Surgery | n=96  Median age 55.5 (range, 18-74) | NCI-CTCAE ver .3.0  EORTC QLQ-C30 ver. 3.0 | Pre-operative baseline  Post-operative weekly for 6 weeks | 14 | - |
| Denis et al. (2014) [39] | France | unknown | Quasi-experimental study | Web-based | No name 3 | Lung cancer  Surgery | n=42 | Weight reporting  5 symptoms on a 0-3 scale  4 symptoms on Y/N | Weekly, between planned visit and imaging follow-up | 14 | - |
| Denis et al. (2019) [40] | France | Unknown | Quasi-experimental study  Feasibility study | Web-based | No name 4 | Solid or hematologic cancer  Chemotherapy | n=41  Median age 60 (range, 44-85) | Body temperature  Reporting one of 6 symptoms | Daily, in between the 5th day of the first cycle of chemotherapy and 3 weeks after the last chemotherapy | 16 | - |
| Falchook et al. (2016) [51] | USA | Unknown | Quasi-experimental study  Feasibility study | Mobile-based | No name 5 | Head & neck cancer  Radiotherapy | n=22  Age<60 years old: n=13 (59%)  Age≥60 years old: n=9 (41%) | Reported Symptom Monitoring System: 5-domain on a 5-tier scale | Once daily, for approximately 5-7 weeks (during the radiotherapy) | 15 | - |
| Furlong et al. (2019) [52] | Austria, Greece, Ireland, Norway, UK | tertiary cancer center | Quasi-experimental pilot study  Feasibility study  *Pilot test for the eSMART RCT (ongoing) | Mobile-based | ASyMS (Advanced Symptom Management System) | Breast, colorectal, Hodgkin's or non-Hodgkin's lymphoma  Chemotherapy | n=62 | Chemotherapy Toxicity Self-Assessment Questionnaire: 10 symptoms + new symptoms | Once daily, for the 1^st^ cycle of the chemotherapy | 13 | - |
| Garcia et al. (2019) [41] | USA | tertiary cancer center, outpatient | Quasi-experimental study | Web-based  Electronic Health Records/Patient Portal integrated | ePRO Screener | Any type of cancer  Unknown | n=3521  Hematologic n=1057, breast n=545  Mean age 57.15 (SD 13.39) | PROMIS Anxiety, Depression, Pain Interference, Fatigue, and Physical Function CATs  Single-item checklists to assess psychosocial and nutritional concerns and needs | Before patients’ scheduled appointments | 12 | - |
| Graetz, Anderson et al. (2018) [42] | USA | tertiary cancer center | RCT  Feasibility study | Web-based | Patient Care Monitor | Gynecologic cancer  Surgery | n=23  control group n=11, intervention group n=12  Control group: app only  Intervention group: app + reminder  Mean age 54.6 | Reporting one of 12 symptoms or no symptom  SF-12 (12-item) at baseline and at the end of study | Once daily  4 weeks | 13 | 15 |
| Graetz, Mckillop et al. (2018) [43] | USA | tertiary cancer center | RCT  Feasibility study | Web-based | Patient Care Monitor | Breast cancer  Chemotherapy | n=43  control group n=22, intervention group n=21  Control group: app only  Intervention group: app + reminder  Mean age 59.9 | FACT-ES: 18-item, on a five-point Likert scale  MMAS-4: 4-item | Twice: baseline, 6-8 weeks later | 14 | 16 |
| Greer et al. (2020) [53] | USA | tertiary cancer center | RCT | Mobile-based | No name 6 | Any type of cancer  Oral chemotherapy | Mobile app intervention group n=91  Control group: standard of care  Intervention group: Mobile ePRO app  Mean age 52.85 (SD 13.74), 92.3% White, 33% hematologic malignancies | MD Anderson Symptom Inventory (MDASI): 19-item, 0-10 scale | Once a week  12 weeks | 12 | - |
| Gustavell et al. (2019) [54] | Sweden | tertiary cancer center | Quasi-experimental study  Feasibility study | Mobile-based | Interaktor | Pancreas cancer  Surgery | n=6  Mean age 65 (range, 57-74) | Reporting one of 12 symptoms with occurrence and frequency | Once daily  4 weeks | 15 | 18 |
| Hauth et al. (2019) [27] | Germany | unknown | Quasi-experimental study  Feasibility study | Web-based | PROMetheus | Pelvic, thoracic, head & neck, upper GI  Radiotherapy | n=21  Pelvic (n=10), thoracic (n=5), head and neck (n=4), upper GI (n=2)  62% were stage 3  Median age 59.4 (IQR 51.5-66.5) | PRO-CTCAE: 24-27 questions (varies from the type of cancer), on a 5-tier scale | Whenever desired (at least once a week) | 10 | - |
| Iivanainen et al. (2019) [35] | Finland | two tertiary cancer centers, outpatient setting | Quasi-experimental study  Feasibility study | Web-based | Kaiku | Immuno-oncological therapy | n=37  Median age 61  64.9% (24/37) male | 18 questions about symptoms  QLQ-30: Quality of life | Every 3 days to weekly  24 weeks | 12 | - |
| Innominato et al. (2018) [44] | France | tertiary cancer center | Quasi-experimental pilot study | Web-based | InCASA with SARA software | Colorectal, pancreatic cancer  Chemotherapy (at home) | n=11﻿  Median age 60 (range, 48-72) | MDASI: 19 items | Once daily  30 days | 14 | - |
| Judson et al. (2013) [36] | USA | tertiary cancer center, outpatient clinic | Quasi-experimental study  Feasibility study | Web-based | STAR | Lung, gynecologic, breast, genitourinary cancer  Chemotherapy | n=286  Median age 58 (range, 30-85) | 7 questions about symptoms  EuroQoLEQ-5D: 5-item | Whenever desired  Average 34 weeks (range 2-214) | 15 | - |
| Langius-Eklof et al. (2017) [55] | Sweden | two university hospitals (one in urban, one in rural area) | Quasi-experimental study | Mobile-based | Interaktor | Prostate cancer  Radiotherapy | n=66  Mean age 69 (SD 5.8) | 14 questions + Open comment section | Once or more daily,  56-77 days (varies from the regimen) | 15 | 20 |
| Lucas et al. (2017) [37] | USA | ﻿5 Community practices (Michigan Urological Surgery Improvement Collaborative (MUSIC)) | Quasi-experimental study  5 pilot practices | Web-based | MUSIC PRO | Prostate cancer  Surgery | n=605 | MUSIC PRO: 21-item | Pre-operative baseline  Post-operative: 3,6,12,24 month | 13 | - |
| Min et al. (2014) [56] | South Korea | tertiary cancer center | Quasi-experimental study  Feasibility study | Mobile-based | Pit-a-Pat | Breast cancer  Chemotherapy | n=30  ﻿mean age 45 years, SD 6; range 36-65 years | Sleep disturbance: 6-item, on a 10-point scale  Quality of total sleep: 10-point scale as a distress thermometer  Mood: 7-point as face expressions  Acute symptoms related to the chemotherapy | Sleep disturbance and mood: twice a day  Acute symptoms: whenever desired  90 days | 15 | - |
| Mooney et al. (2017) [58] | USA | 4 tertiary cancer centers + 2 public hospitals | RCT | Interactive Voice Response (IVR) | SCH (Symptom Care at Home) | Any type of cancer  Chemotherapy | n=358  Control n=178, intervention n=180  Control: captured data were not seen by a nurse practitioner  Intervention: captured data were seen by a nurse practitioner  Mean age 55.77 (SD 11.42)  - control: 56.79 (SD 10.54)  - intervention: 54.77 (SD 12.77) | Reporting one of 11 symptoms, severity on a 0-10 scale | Once daily  Average 77 days | 14 | - |
| Mougalian et al. (2017) [59] | USA | tertiary cancer center | Case-controlled pilot feasibility study | Bidirectional Text Messaging | BETA-Text (Breast Cancer Endocrine Therapy Adherence-Text) | Breast cancer  Endocrine therapy | n=100﻿  Historically control group was used as BETA-Text non-user group (n=100)  stage I to III HR-positive breast cancer on advanced endocrine therapy  median age 53.5 (IQR 47-62) | 3 symptoms related to the treatment, on a 1-9 scale  Adherence survey: 4-item, on a 4-point Likert scale | Weekly for symptoms, monthly for adherence  3 months | 15 | - |
| Peltola et al. (2016) [28] | Finland | tertiary cancer center | Quasi-experimental pilot study  Feasibility study | Web-based | Kaiku | Head and neck cancer  Radiotherapy | n=5  age range 50-80 | CTCAE ver. 4.03: items vary on the type of cancer, on a 1-4 scale  15D: 15-item  EORTC QLQ-H&N35: 35-item | Weekly during the treatment and a month after the completion of treatment | 10 | - |
| Snyder et al. (2013) [45] | USA | tertiary cancer center | Quasi-experimental study  Feasibility study | Web-based | PatientViewpoint | Breast, prostate cancer  Unknown | n=47  median age 58 (range, 28-81) | PROMIS 6 domains  EORTC-QLQ BR23 for breast cancer  EPIC short form for prostate cancer | Every 2 weeks | 15 | - |
| Sundberg et al. (2015) [57] | Sweden | two university hospitals (one in urban, one in rural area) | Quasi-experimental study  Feasibility study | Mobile-based | ICT-Platform (pilot version of Interaktor) | Prostate cancer  Radiotherapy | n=10  mean age 69 | Reporting one of 13 symptoms | Once daily  2 weeks | 12 | 18 |
| Warrington et al. (2019) [46] | UK | tertiary cancer center | Quasi-experimental study  Feasibility study | Web-based | eRAPID | Breast cancer  Chemotherapy | n=12  ﻿mean age=47.5 years (SD 10.3), range 33–73 years | CTCAE 12 core items + free text option | Once a week  12 weeks | 14 | 17 |
| Zylla et al. (2019) [47] | USA | tertiary cancer center | Quasi-experimental study  Feasibility study | Web-based  Electronic Health Records/Patient Portal integrated | No name 7 | Non-hematologic cancer | MyChart group n=45  3 groups  - Paper  - Phone Call  - MyChart  median age in MyChart group 62 (range, 39-84) | PRSM: 23 questions | Every 2 weeks  12 weeks | 13 | - |
