# Supplementary figures and images for: Acceptance and Use of Home-Based Electronic Symptom Self-Reporting Systems in Patients With Cancer: Systematic Review
Source: J Med Internet Res. 2021 Mar 12;23(3):e24638. doi: 10.2196/24638 (PMC7998328; doi:10.2196/24638)

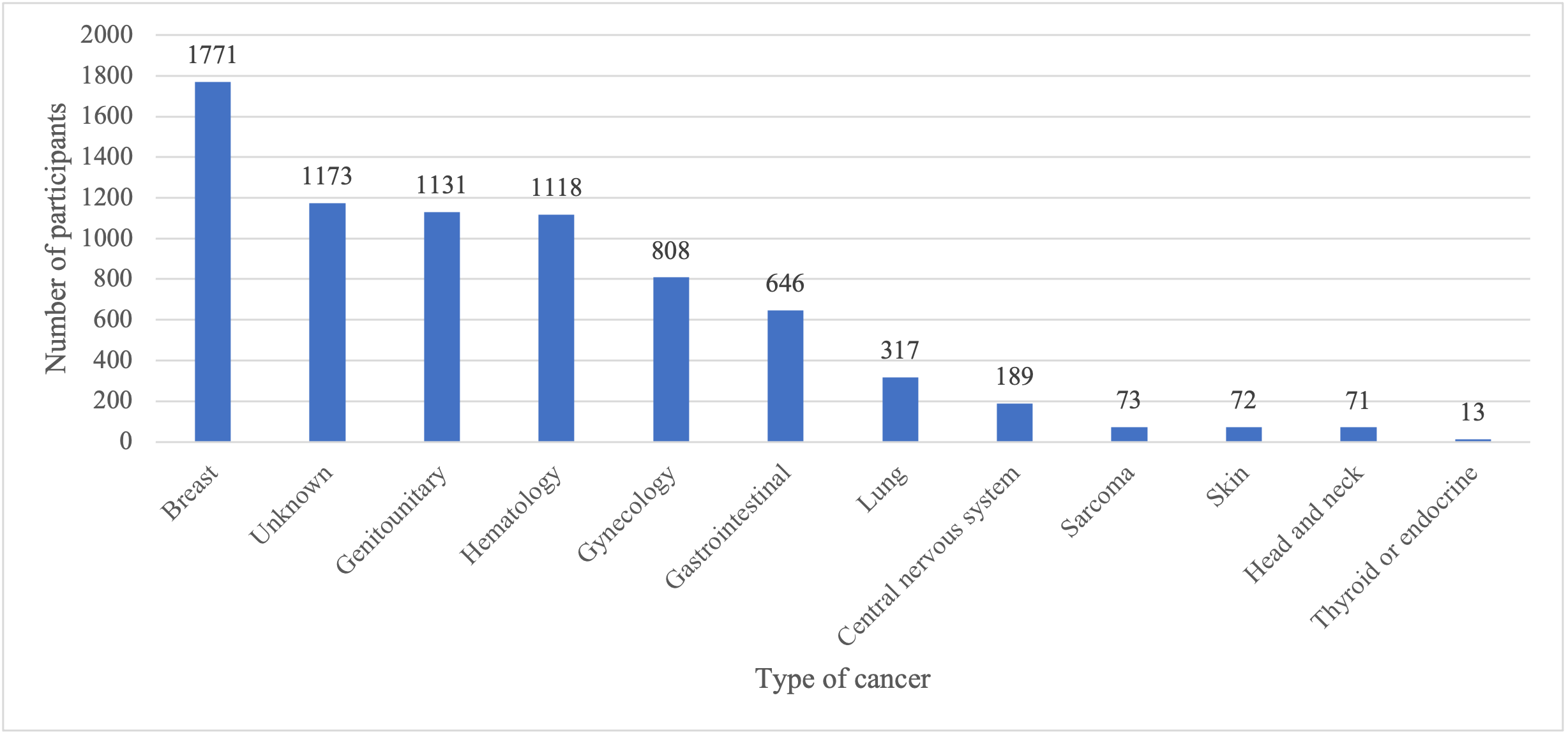

Supplement: Multimedia Appendix 3 [file jmir_v23i3e24638_app3.png]
