# Supplementary material for: Acceptance and Use of Home-Based Electronic Symptom Self-Reporting Systems in Patients With Cancer: Systematic Review
Source: J Med Internet Res. 2021 Mar 12;23(3):e24638. doi: 10.2196/24638 (PMC7998328; doi:10.2196/24638)
Supplement: Multimedia Appendix 4 [file jmir_v23i3e24638_app4.docx]

Table 3. Measures of patients’ use of e-SRS in studies

| **Calculations of Measures** | | **Referenced Studies** | **Range of Use** |
| --- | --- | --- | --- |
| **Denominator** | **Numerator** |  |  |
| The number of all enrolled patients | The number of patients who submitted forms (symptoms) | [27,47–49,50] | 56-95% |
|  | The number of patients who submitted symptoms at least times  | [31,38,41,46,48] | 9-85.1% |
|  | The number of patients enrolled during specific timeframes | [31,34–36,45,59] | 62-95% |
|  | The number of forms (symptoms) submitted | [53] | 14.70 forms |
|  | The number of patients who logged on to systems | [30] | 99% |
|  | The number of patients who logged on to systems at least times  | [30,34,45,46] | 33-77% |
|  | The number of logging-on to systems | [42] | 4.0 times |
|  | The number of days that each patient logged-on to systems | [53] | 21.76 days |
| The number of patients who submitted forms (symptoms) | The number of forms (symptoms) submitted | [35] | 11 forms |
| The number of patients who logged on to systems | The number of log-ons | [36] | 17 times |
| The number of all expected forms (symptoms) | The number of forms (symptoms) submitted | [37,39,40,44,35,52,59] | 67.4-88.4% |
| The number of weeks in study period | The number of forms (symptoms) submitted | [43,46] | 55-63% |
| The number of days in study period | The number of days with any patient submitted forms (symptoms) | [44,51,56,52] | 45-90% |
|  | The number of forms (symptoms) submitted | [54,55] | 84-87% |
